# Bioactive Molecules for Discriminating *Robinia* and *Helianthus* Honey: High-Performance Liquid Chromatography–Electron Spray Ionization–Mass Spectrometry Polyphenolic Profile and Physicochemical Determinations

**DOI:** 10.3390/molecules26154433

**Published:** 2021-07-22

**Authors:** Otilia Bobiş, Victoriţa Bonta, Mihaiela Cornea-Cipcigan, Gulzar Ahmad Nayik, Daniel Severus Dezmirean

**Affiliations:** 1 Department of Beekeeping and Sericulture, University of Agricultural Sciences and Veterinary Medicine of Cluj-Napoca, 400372 Cluj-Napoca, Romania; victorita.bonta@usamvcluj.ro; 2 Department of Horticulture and Landscaping, University of Agricultural Sciences and Veterinary Medicine of Cluj-Napoca, 400372 Cluj-Napoca, Romania; mihaiela.cornea@usamvcluj.ro; 3 Department of Food Science & Technology, Govt. Degree College Shopian, Srinagar 192303, India; gulzarnaik@gmail.com

**Keywords:** honey, *Robinia*, *Helianthus*, flavonoids, phenolic acids, biochemical markers, chromatography

## Abstract

Bioactive molecules from the class of polyphenols are secondary metabolites from plants. They are present in honey from nectar and pollen of flowers from where honeybees collect the “raw material” to produce honey. *Robinia pseudoacacia* and *Helianthus annuus* are important sources of nectar for production of two monofloral honeys with specific characteristics and important biological activity. A high-performance liquid chromatography–electro spray ionization–mass spectrometry (HPLC–ESI–MS) separation method was used to determine polyphenolic profile from the two types of Romanian unifloral honeys. *Robinia* and *Helianthus* honey showed a common flavonoid profile, where pinobanksin (1.61 and 1.94 mg/kg), pinocembrin (0.97 and 1.78 mg/kg) and chrysin (0.96 and 1.08 mg/kg) were identified in both honey types; a characteristic flavonoid profile in which acacetin (1.20 mg/kg), specific only for *Robinia* honey, was shown; and quercetin (1.85 mg/kg), luteolin (21.03 mg/kg), kaempferol (0.96 mg/kg) and galangin (1.89 mg/kg), specific for *Helianthus* honey, were shown. In addition, different phenolic acids were found in *Robinia* and *Helianthus* honey, while abscisic acid was found only in *Robinia* honey. Abscisic acid was correlated with geographical location; the samples collected from the south part of Romania had higher amounts, due to climatic conditions. Acacetin was proposed as a biochemical marker for Romanian *Robinia* honey and quercetin for *Helianthus* honey.

## 1. Introduction

The composition of honey comprises mainly simple sugars and water. However, more than 200 other constituents, such as enzymes, amino acids and organic acids, carotenoid-like substances, Maillard reaction products, vitamins, minerals and polyphenols, are present in honey [1]. Most of these compounds represent bioactive molecules, giving the biologically active properties of honey.

From the numerous compounds of honey, nowadays it is known that some of them act similar to antioxidants: vitamin C, vitamin E, enzymes (catalase, peroxidase) and phenolic compounds [2,3,4,5,6].

Phenolic phytochemicals are important aromatic secondary metabolites in plants [7,8], found as single aglycones or bonded with different sugars (glucose, arabinose, galactose and rhamnose), forming the flavonoid glycosides. Several studies have been carried out, over time, on many types of honey, and different polyphenolic compounds have been assessed as markers for authenticity and botanical origin determination [9,10,11].

The botanical origin of monofloral honey is mainly performed by pollen analysis, which evaluates correctly the presence of specific pollen grains in honey sediment. In addition, the profile of flavonoids and phenolic acids was used in the last years for this classification. This is an important issue because many monofloral honeys have increased prices and also limited production and availability, with this being the reason that they are more often subjected to falsification.

The flavonoids apigenin, quercetin, luteolin, kaempferol and galangin, reported in honey [12,13,14,15], and pinocembrin, pinobanksin and chrysin derived from propolis, were determined in many European monofloral honeys [3,9,16].

Honey biological activity is associated with the presence of phenolic compounds (phenolic acids and flavonoids), but also to other different phytochemicals, such as ascorbic acid, amino acids and different proteins, and also the presence of H_2_O_2_ [4,17,18,19,20]. Manuka honey, for example, has as bioactive markers of its antioxidant and antibacterial properties methyl syringate and leptosin [21]. 

The available scientific literature indicates increasing published research data regarding the chemical composition, bioactivity, the profile and content of flavonoids and phenolic acids in Romanian honeys [17,20,22,23,24,25,26,27,28,29,30,31,32]. This is due to the variety and quality of Romanian honey, the fact that agriculture is less developed and chemicalized, and the fact that different landscapes provide very good “raw material” for the bees (nectar, pollen and honeydew), materials clean of any contaminants and having a huge variety of botanical origins. Flower nectar and pollen and also honeydew are known as “raw materials” from which the bees are making honey. These materials need to be free of any contaminants to have a high quality honey, which, in Romania, may happen as a result of the reasons stated before. Therefore, the main objective of this work was to identify and quantify the content of these phytochemicals as possible biochemical markers for two types of Romanian floral honeys (*Robinia* and *Helianthus*) and to compare with those reported for the same honey types from Europe. We chose these honey types due to their market demands outside of Romania (Black locust honey) [24,33] and the high bioactive properties of sunflower honey both for humans and for bees, as demonstrated in different other studies [34,35].

## 2. Results and Discussions

Generally, in honey, phytochemicals are present as polyphenols (mostly phenolic acids and free flavonoids). In the last decades many literature data regarding the content of polyphenolic content of different types of honey all over the world, including Romania are available [3,9,11,12,13,14,15,18,19,20,27,36,37,38,39,40,41].

### 2.1. Quality General Parameter Results for Investigated Honey Samples

Honey samples from different regions from Romania were collected directly from beekeepers. From the honeys collected for the study, only ten *Robinia* and eight *Helianthus* were confirmed as monofloral honeys following palynological analysis. The pollen content of the authentic honeys varied between 21 and 36% for *Robinia pseudoacacia* honey and between 38 and 52% for *Helianthus annuus* honey (in accordance with Romanian standard SR 784/1-3/2009 [42] and Persanno Oddo and Piro (2004) [43]. Mean value of *Robinia* pollen grains was 29.2% and *Helianthus* pollen grains was 46.5%, these values being in accordance also with the descriptive sheets of unifloral honeys [43]. Romanian standard (SR 784/1-3/2009) states a minimum of 25% *Robinia*-specific pollen in the sediment of *Robinia* honey, to be considered as monofloral, and a minimum of 30% sunflower pollen, to be considered *Helianthus* monofloral honey.

The results of the main physicochemical characteristics for the black locust (*Robinia pseudoacacia*) and sunflower (*Helianthus annuus*) honey samples are presented in Table 1.

The results demonstrated that the tested honey samples can be regarded as monofloral type of *Robinia* and *Helianthus* honeys. The moisture content of analyzed samples ranged between 17.3 and 19.0% (Table 1); all samples meet the criterion established by Codex Alimentarius standard (<20%) [44]. Different factors are responsible for moisture content of honey: ripening process in the hive, extraction, processing and storage of honey, as well as the humidity of the region where honey is produced [20]. Lower moisture content was determined in black locust honey samples (average of 17.9%), compared to sunflower (average of 18.2%). This difference is not significant; botanical origin is not a determinant factor for this parameter. The nectar flow may be the reason for different results obtained for the two honey types, and the ripening period maintained by the beekeepers until they harvest the honey. As long as the values do not overcome the standard limit (20%), they are declared conform and no danger of fermentation is present. The obtained values are comparable with other studies on honey from Romania [17,20,22,23,26,30,31,32]. Electrical conductivity is another parameter used in honey authentication; monofloral light honeys must have low values of this parameter. Although this is a parameter used for honeydew determination, where higher values than 0.700 mS/cm are requested, it may be used also for floral honey analysis. Black locust honey analyzed in this study presented an average value of 0.155 mS/cm, and 0.473 mS/cm was for sunflower honey. These values are in accordance with different standard regulations (<0.500 mS/cm for pure floral honeys). The HMF content is a parameter indicating the freshness of honey, the authenticity, a possible thermal treatment or inadequate storage conditions [20]. It is present in fresh honey in small amounts; its value is correlated with harvest ambient temperature, storage conditions and processing. European legislation [45] allows for a maximum content of 40 mg/kg HMF, but the analyzed samples present a much lower amount (average of 1.39 mg/kg for black locust honey and 4.35 mg/kg for sunflower honey). Other studies on black locust and sunflower honey reported similar values [17,20,22,27,28,30,33,46]. Honey main components of the chemical composition are simple sugars and water. Monosaccharides (fructose and glucose) represent 65–75% from the total soluble solids. Black locust honey is a special type of honey due to the high content of fructose (generally >40%), which maintains this type of honey in liquid state for a long period of time. From the analyzed *Robinia* samples, 80% presented fructose content over 40%, with the highest value being 45.99% (sample RH-07). On the other hand, *Helianthus* honeys presented lower values of fructose (below 39.49%). Regarding the glucose content, *Robinia* honey presented an average value of 31.02%, with more than 10% lower than fructose. For *Helianthus* honey, an average of 42.33% glucose was registered in the analyzed samples. Sucrose is the disaccharide present in honey as residue from the flower nectar, where it is present in higher amounts and from where the bees are converting in fructose and glucose with the help of amylase. Standard regulations allow a maximum content of 5% sucrose; a higher value is regarded as a possible adulteration. 

The fructose content of analyzed black locust honey ranges between 38.67 and 45.99%, and glucose content ranges between 28.86 and 33.05%. Sunflower honey presented higher values for glucose content (average 42.33%) compared to fructose (average 38.83%). The sucrose content for all the analyzed samples shows values below 2%, indicating the honeys’ authenticity—no suspicion of adulteration with sugar syrup being present.

An important parameter for honey botanical origin determination is fructose/glucose (F/G) ratio, which must be higher than 1.4 for *Robinia* honey, to maintain this type of honey in liquid state for a longer period of time. This ratio was obtained for most of the analyzed *Robinia* samples (Table 1). Instead, the other types of honey, which crystalize more easily and in a shorter period of time, are characterized by a more equilibrate fructose and glucose content. 

Sunflower honey is characterized by a F/G ratio below 1 (in this type of honey the crystallization process occurs in few weeks after harvesting) (Table 1). 

Similar values for physicochemical parameters were obtained for Romanian black locust and sunflower honeys [17,20,22,23,27,28,31,32] analyzed from different locations in Romania and also honeys from different geographical locations (Serbia or Italy) but of the same botanical origin [47,48]. 

Several statistical methods were performed to make different correlations between the honey samples and explain their composition. On the basis of the Principal Component Analysis (PCA), the first two principal components explained 94% of the data variance, showing a good discrimination of the honey samples.

**Figure 1:**
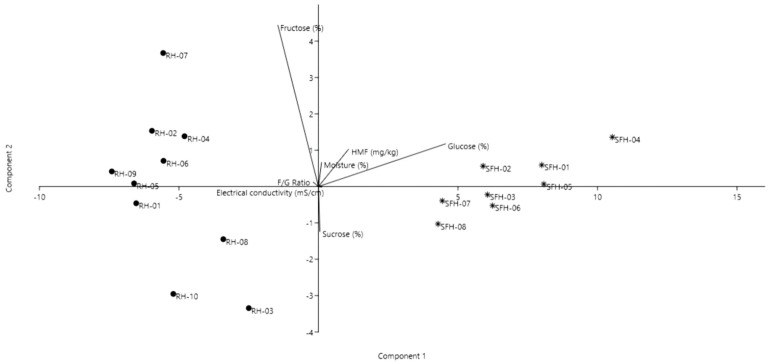
Principal component analysis biplot of the honey samples based on the physicochemical analysis.

As shown in Figure 1, the analyzed samples were clearly differentiated based on honey type and physicochemical analysis. Thus, in the first upper quadrant, the samples rich in fructose and F/G ratio, as well as the moisture content, are displayed. In the second quadrant, samples RH-03, RH-08 and RH-10 are differentiated from the others having higher electrical conductivity and higher values in glucose and sucrose. In the following third and fourth quadrants, the samples SFH-03, SFH-06, SFH-07 and SFH-08 had higher electrical conductivity and sucrose content, whereas the sunflower samples had a high sucrose and F/G ratio content, as well as high values in fructose and HMF. Overall, the black locust samples were distinct due to their higher fructose content and, consequently, F/G ratio, whereas the sunflower honey samples had high values of glucose, moisture and HMF.

### 2.2. Total Polyphenolic and Flavonoid Content

To determine the total phenolic and flavonoid content, honey samples were dissolved in methanol and a mixture of methanol:water (pH = 2), 1:1 (*v/v*), as described in the Material and Methods section. As observed in Table 2, higher values of total phenolics in black locust honeys were determined when methanol was used as solvent for honey preparation (75.55–85.44 mgGAE/100 g honey). Using the mixture of methanol and water (1:1, *v*/*v*), the total phenolic content was lower, but not statistically different (60.19–79.77 mgGAE/100 g honey). Sunflower honey presented a higher content of total polyphenols, again with higher amounts when methanol was used as solvent (92.40–134.21 mgGAE/100 h honey), compared to methanol:water as solvent (88.65–107.55 mgGAE/100 g honey). Different studies in the literature present extremely large variations in the total polyphenolic profile of black locust and sunflower honeys. This observation was made for honey samples from Romania previously analyzed [17,20,27], as well as samples from Poland that were recently characterized [3,5]. A study of Mărghitaş et al. (2009) [17] found in Romanian black locust honey samples, a total polyphenolic content of 2.00–39.00 mgGAE/100 g honey. In the same range was situated a study on *Robinia* honey from Romania from 2014 [23], where an average of 18.40 mg/100 g honey total phenols was obtained. Ciucure and Geană (2019) [27] quantified higher amounts of total phenolic content in pure *Robinia* honeys from Romania (31.20–80.40 mgGAE/100 g honey). The amount of total phenolics from our study was in accordance with the last mentioned study [27]. In Poland, Halgarada et al. (2020) [3] performed a study on several organic and conventional honeys. Following the palynological origin determination, not all declared botanical origins of the honeys were certified. For example, two samples declared as *Robinia* honey, did not have any *Robinia* pseudoacacia pollen in the sediment, being declared multifloral and rapeseed honeys. These honeys were classified as light-colored honeys, and their total phenolic content certified this (4.15 mgGAE/100 g honey). Another study made on Polish honeys, performed recently [5], found very high amounts of total polyphenols (average of 76.30 mg/100 g honey) in *Robinia* samples; this is also in accordance with our studies. As specified before, sunflower honey presented a higher total phenolic content when methanol was used as solvent in sample preparation, but again with no statistical significance compared to water:methanol mixture. For sunflower honey, the results obtained by different authors are more homogenous, compared to *Robinia* honey, but still large variations were obtained. Total phenolic content of sunflower honeys from Romania was between 20.00 and 45.00 mgGAE/100 g honey in a study from 2009 [17]. Another study from 2015 [49], on Romanian sunflower honey, found an average of 84.51 mgGAE/100 g honey of total phenolics, compared to a recent study [20], where only 21.1 mgGAE/100 g honey was quantified. Using two types of solvents for dissolving honey samples, the total phenolic content in our samples was higher than the mentioned studies of Romanian sunflower honeys (average of 95.50 mgGAE/100 g honey in water:methanol, and 106.64 mgGAE/100 g honey in methanol. 

**Figure 2:**
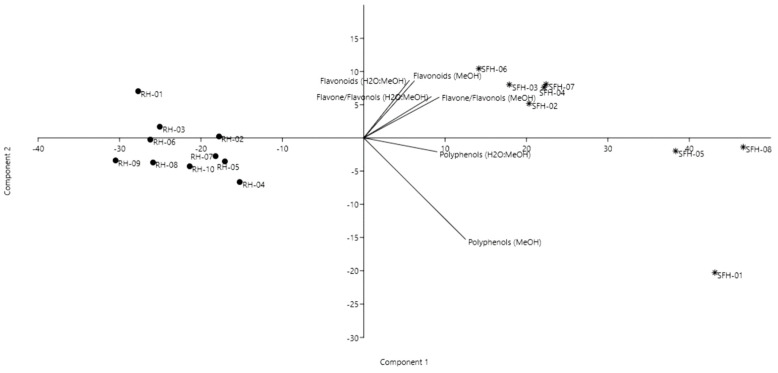
Principal component analysis biplot of the honey samples based on total polyphenols and flavonoid content.

For the flavonoid content, two methods were performed to quantify flavone/flavonol and total flavonoids content. Using the two mentioned solvents for honey preparation, we obtained different results (Table 2). While for the flavone content determination, the best solvent was methanol, where higher amounts of flavone were obtained, for the total flavonoid content, a higher amount was quantified when the honey was dissolved in the mixture of methanol and water. A similar discussion as for total phenolics is valid also for flavonoids. Our results are in accordance with previous studies on honey from Romania [49] and Poland [3]. In order to see if the samples are grouped also according to the polyphenolic and flavonoid content, a second PCA was performed to emphasize whether there are significant correlations between the honey types. Furthermore, we wanted to highlight if there are significant differences between the extraction solvent. A good discrimination of the honey samples was achieved (Figure 2), with the first and second components explaining 96% of the total variance.

Black locust and sunflower honeys were clearly differentiated. No significant difference was noticed in the different extraction solvent except in terms of polyphenols, especially in samples SFH-01, SFH-05 and SFH-08, which displayed higher values compared to the other sunflower samples. 

Black locust honeys had similar polyphenol and flavonoid content, except for sample RH-01, followed by RH-02, which displayed higher total flavonoid content in the water:methanol extract. Our results are in accordance with Lazarević et al. (2012) [47], which demonstrated the relationships between the physicochemical parameters and the botanical origin of black locust, sunflower and linden honeys. Their results showed that predictive models, such as PCA, could be successfully used to distinguish between different honey types.

### 2.3. Antioxidant Activity

Black locust honey is a light-colored honey with low amount of bioactive constituents; it also has low amounts of enzymes, minerals and pigments compared to other honey types [17,18,23,26,27,30,34,39,43]. Sunflower honey is richer in bioactive compounds [17,20,31,32,39,43]. This was confirmed again following this study (Table 3); higher radical scavenging activity for sunflower honey, as well as the lower IC_50_, compared to black locust honey, was obtained. 

As seen in Table 3, significantly, the highest inhibitory activity was noticed in sunflower honey samples SFH-05 (5.98 mmol Trolox/100 g), followed by SFH-08, whereas, in the black locust samples, the highest level of inhibition was observed in RH-04 (4.80 mmol Trolox/100 g), followed by RH-02 and RH-07 The lowest radical scavenging activity in sunflower honey was determined in SFH-02 (5.11 mmol Trolox/100 g); in black locust honeys, the lowest value was determined in RH-05 sample (3.97 mmol Trolox/100 g). Low radical scavenging activity was obtained for black locust and sunflower honeys from Romania in this study, similar to same honeys of different geographical origin [39]; however, a high inhibition percent was obtained for sunflower honey from Romania by Pauliuc et al. (2020) [20]. Low scavenging activity was also obtained for other light honeys from Poland (linden and Canola) [50], with an average of 12.4–13.6% inhibition. 

Similarly, the strongest reducing antioxidant power measured by the FRAP assay was found in the aforementioned sunflower honey samples SFH-05 (1.398 mM Fe^2+^) and SFH-03 (1.249 mM Fe^2+^). In the black locust honeys the strongest activity was noticed in samples RH-07 (0.793 mM Fe^2+^), followed by RH-10 (0.770 mM Fe^2+^). The lowest reducing antioxidant power was observed in SFH-07 (0.972 mM Fe^2+^) and RH-05 (0.652 mM Fe^2^). 

### 2.4. LC–MS Analysis of Phenolic Acids and Flavonoids from Robinia Pseudoacacia Honey

One important problem in the analysis of flavonoids from honey is the high sugar content. For this reason, a complex sample preparation, using a non-ionic polymeric resin (Amberlite), is generally used. Using this method of sample preparation, a good recovery and reproducibility of phenolic compounds is obtained [3,9,11,13].

The phenolic profile separated and identified by HPLC–ESI–MS from *Robinia* honey (example Supplementary Materials Figure S1; sample RH-06) consist of 3 phenolic acids (*p*-hydroxybenzoic, ferulic and *t*-cinnamic acid), a non-phenolic compound (abscisic acid) and 5 free flavonoids (pinobanksin, apigenin, pinocembrin, chrysin and acacetin).

The total content of identified phenolic acids was situated between 1.33 and 19.56 mg/kg honey, with ferulic acid being identified in all analyzed samples (0.72–8.66 mg/kg). A higher quantity of *p*-coumaric acid was obtained, but only in 33% of the analyzed samples (Table 4).

The other phenolic acid profile of *Robinia* honey includes *p*-hydroxybenzoic, *t*-cinnamic and vanillic acid quantified in different amounts (average of 0.26, 0.62 and 0.72 mg/kg). These phenolic acids were not found in all analyzed samples (Table 4). From samples with similar botanical origin, Tomas-Barberan et al. (2001) [9] identified also ferulic acid and *p*-coumaric acid in samples originating from Germany and Italy. More recent studies [3,50] identified similar amounts of ferulic, vanillic, *p*-coumaric and caffeic acid in black locust honeys from Poland. 

All tested samples contain pinobanksin (0.85–2.28 mg/kg), pinocembrin (0.38–1.48 mg/kg) and chrysin (0.69–1.24 mg/kg). These flavonoids originating from propolis were also described in previous studies [9], as well as in more recent ones [3]. 

The amounts quantified in our studies were although smaller than in the mentioned literature, but higher than in other studies analyzing Croatian *Robinia* honey [13]. High differences between the amounts of different phenolics for *Robinia* honey is observed in the samples originating from different geographic areas and not between the profiles of these compounds. Different reasons for this observation, such as climatic conditions, the purity of the sample, the method of analysis and the sensitivity of the apparatus, could be taken into consideration to explain such varieties in the amounts of identified and quantified phytochemicals.

It is known that polyphenols are secondary plant metabolites with protective function on plant survival under different environmental stress conditions and pathogenic attacks [51]. According to different reports in the literature, the production or accumulation of different phenolics in plants is related to temperature, soil, light and water, and it is demonstrated that the amount of these compounds is related to these factors. The synthesis of flavonoids was previously proposed as a plant defense mechanism against stress, and different geographical and climatic conditions may influence greatly the quantity of these compounds in plants, flower pollen and also in honey [52].

A similar profile for individual phenolics was also evidenced by Tomas Barberan et al. (2001) [9] and Goślinski et al. (2021) [39]; not all phenolics were identified and quantified in all analyzed samples of the same botanical type. 

Different studies report acacetin as abundant component in *Robinia* honey samples [22,48]. In all analyzed samples originating from Romania, this flavonoid is also identified. The amount of this compound varied between 0.49 and 2.24 mg/kg honey. After comparing the retention time, UV spectra and MS fragmentation with an authentic standard, we proposed this compound as biochemical marker for *Robinia* honey.

The total amount of identified flavonoids in *Robinia* honey varied between 3.75 and 8.04 mg/kg honey, with pinobanksin, acacetin and apigenin quantified in amounts, representing 25.6, 19.1 and 15.9% from the total amount of identified flavonoids and a lower content for the rest of flavonoids (chrysin, kaempferol and pinocembrin) as 8.7–15.3% from total identified flavonoids (Table 4).

### 2.5. Abscisic Acid in Robinia Pseudoacacia Honey

A high content of abscisic acid (average of 11.24 mg/kg honey) has been quantified in analyzed *Acacia* honey samples. Abscisic acid is not a phenolic acid, but it has very similar behavior, presenting strong UV absorbencies at 248 and 270 nm. This plant hormone is known to be present in floral nectar, and the presence in honey is also expected [9]. It is related to plant protection against dryness and environmental stress. Abscisic acid has been found in many types of honey (heather, rapeseed, lime-tree, *Robinia, Leptospermum* and sunflower) in different amounts [12,53,54]. It was detected in high amounts (13.93–23.03 mg/kg) in the samples collected from the southern part of Romania; we observed that high temperature in the season, as well as dryness (lack of humidity), was a determinant for the quantified amounts, while samples collected from inside the Transylvania (hilly region, with more humidity; presented in Material and Methods section), presented lower amounts of abscisic acid (2.95–6.61 mg/kg). This observation demonstrates once again that its presence in honey is related to plant protection against different stressors, with the samples being harvested in different geographical areas, with different soil substrates and variable climatic conditions.

### 2.6. LC–MS Analysis of Phenolic Acids and Flavonoids from Helianthus Annuus Honey

The LC–MS chromatogram of *Helianthus annuus* honey (Supplementary Materials Figure S2, exemplified by sample SFH-02) shows a profile of 12 phenolics: four phenolic acids (protocatechuic, chlorogenic, caffeic and *p*-coumaric acid) and eight free flavonoids (quercetin, luteolin, pinobanksin, apigenin, kaempferol, pinocembrin, chrysin and galangin). Analyzed sunflower honey samples were more homogenous regarding the phenolic profile, with most of the compounds being identified in all analyzed samples. 

The quantified phenolic acid profile was very similar (mean quantities of 1.97 to 2.84 mg/kg honey) for all compounds. The phenolic acids present in all analyzed samples were protocatechuic acid (1.98–3.74 mg/kg), chlorogenic acid (1.63–2.37 mg/kg), caffeic acid (2.08–4.16 mg/kg), *p*-coumaric acid (1.98–3.85 mg/kg) (Table 5). In 66% of the samples was identified also *p*-hydroxybenzoic acid (1.79–2.07 mg/kg). These amounts were higher than those reported by Thomas-Barberan et al. (2001) [9] and Pulcini et al. (2006) [55] in sunflower honey from France and Italy, for the same phenolic acids (0.9 mg/kg for caffeic acid and 0.27 and 0.78 mg/kg, respectively, for *p-*coumaric acid). 

Caffeic acid was also identified and quantified in honeys originating from Italy and France [9] in quantities approximately two times smaller than in Romanian honey (1.11 mg/kg compared to 2.94 mg/kg). A recent study of different Romanian honeys quantified 0.3 mg/100 g caffeic acid in sunflower honey [20]. 

As common flavonoids, quercetin (1.21–2.33 mg/kg), luteolin (17.5–27.09 mg/kg), pinobanksin (1.56–2.13 mg/kg), kaempferol (0.54–1.29 mg/kg), pinocembrin (1.05–2.18 mg/kg), chrysin (0.56–1.59 mg/kg) and galangin (1.52–2.39 mg/kg) were identified and quantified in Romanian sunflower samples.

Apigenin was quantified in 66% of the analyzed samples (Table 5). As it can be seen from the chromatogram (Supplementary Materials Figure S2) and Table 5, the major flavonoid in the samples was luteolin (representing 67% from the total) and the other flavonoids representing less than 10% from the total identified flavonoids.

The presence of quercetin in all Romanian honey samples confirm this compound as biochemical marker for this type of honey (Supplementary Materials Figure S3), as was stated before by different authors analyzing honey from Romania or other geographical origins [9,20].

Relatively small differences between the content of quercetin from honeys originating from different European countries were reported, the average content of this compound being situated between 1.31 and 2.86 mg/kg honey [9,20,55]. Quercetin in Romanian sunflower honey was situated between 1.21 and 2.42 mg/kg, similar to same botanical origin honey from other Romanian regions, France and Italy.

As an additional biochemical marker for Romanian studied sunflower samples, we propose luteolin, which is present in very high amounts (17.50–27.09 mg/kg), representing 46% from total identified flavonoids. 

Our samples can be divided in two types of honey regarding their constituents: first type is that for which the presence of possible characteristic markers were detected and need to be identified in further studies of honey and pure nectar (i.e., *Robinia* honey), and the second type is that for which floral markers were reported previously and our studies confirm these markers (i.e., *Helianthus* honey).

**Figure 3:**
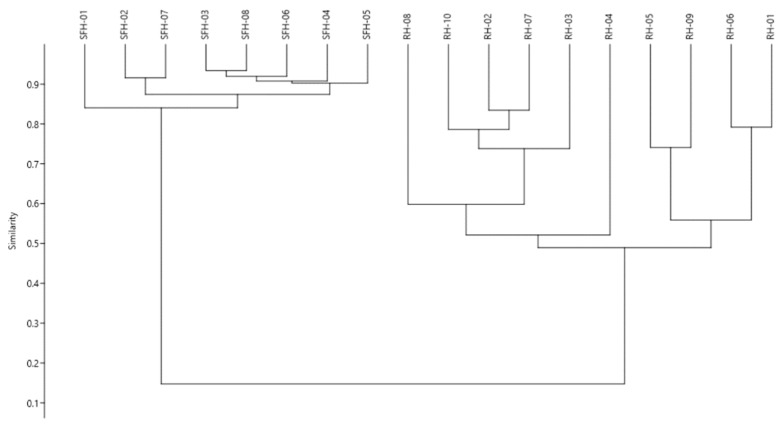
Hierarchical clustering of the honey samples based on the phenolic acids and flavonoids content.

As a part of the statistical interpretation of the results, we performed a hierarchical cluster analysis to verify if the data structure could identify different groups among the honey types. For the analysis, the whole dataset incorporating all honey samples, along with the phenolic acids and flavonoids content, was used, and the paired group (UPGMA) algorithm was applied by using the Bray–Curtis similarity to space the cluster. As it can be foreseen (Figure 3), the hierarchical clustering (*r* = 0.98) organized the samples in two main clusters based on the honey type. The first major branch of the dendrogram includes the sunflower honey samples, with special attention on sample SFH-01 rich in *p*-hydroxybenzoic acid and luteolin and pinobanskin. The following samples (SFH-02 and SFH-07) had the highest content in phenolic acids and flavonoids compared to the others. The next branch comprises the other sunflower samples with lower levels in phenolic acids and flavonoids. The second major branch comprises the black locust honey samples. The first sub-cluster differentiates the sample RH-04 from the others due to its high content in phenolic acids (*p*-coumaric, vanillic, ferulic, *t*-cinnamic and abscisic acids). Furthermore, a high content in kaempferol and acacetin flavonoids was noticed. Finally, the last sub-cluster comprises the samples rich in ferulic acids, as well as in pinocembrin and chrysin. At the same time, low levels in *p*-hydroxybenzoic, *p*-coumaric, *t*-cinnamic and abscisic acids were noticed. 

## 3. Materials and Methods

### 3.1. Chemicals

Standards of flavonoids: Quercetin (3,3’,4’,5,7-pentahydroxyflavone), catechin (3,5,7,3’,4’-pentahydroxyflavan), luteolin (3’,4’,5,7-tetrahydroxyflavone), apigenin (4’,5,7-trihydroxyflavone), naringin (naringenin-7-β-rhamnoglucozid), kaempferol (3,4’,5,7-tetrahydroxyflavone), galangin (3,5,7-trihydroxyflavone), pinocembrin (5,7-dixydroxyflavanone), chrysin (5, 7-dihydroxyflavone), acacetin (apigenin-4’-methylether) and different phenolic acids: *p*-hydroxybenzoic, caffeic, ferulic, chlorogenic, *p*-coumaric, *t*-cinnamic, *o*-coumaric, vanillic, homovanillic, protocatechuic and syringic were acquired from Sigma Aldrich (Saint Louis, MO, US) and Fluka Chemie GmbH (Buchs, Switzerland). Folin–Ciocâlteu reagent (2N), sodium carbonate, 2,2-diphenyl-1-picrylhydrazyl, aluminum chloride, sodium nitrite, sodium hydroxide, Ferrous sulfate, and Trolox (Sigma Aldrich Co.) were used for total phenolics and antioxidant activity determination. Organic solvents (methanol, acetonitrile, ethyl acetate and acetic acid) were analytical, HPLC or MS grade (Sigma Aldrich Co.). Ultrapure water was obtained with a Millipore equipment (MilliQ Integral 3, Millipore, Burlington, MA, USA).

### 3.2. Honey Samples

Honey samples from two declared botanical origins (22 samples of declared black locust honey and 15 declared sunflower samples) were collected from the beekeepers during one beekeeping year (2018) and stored at 4 °C until analysis. Botanical origin was confirmed by the combination of classical quality determinations and pollen analysis. After palynological origin determination, ten samples of black locust honey (five samples originating from beekeepers located in the Transylvanian Plateau (RH-01; RH-05; RH-06; RH-08; RH-09) and five samples from southern part of Romania (RH-02; RH-03; RH-04; RH-07; RH-10) and eight samples of sunflower (obtained from Southern Romania) were subjected to physicochemical determination of the main chemical characteristics and phenolic profile determination. We chose these geographic areas because they are the main places for producing both *Robinia* and *Helianthus* honey.

### 3.3. Physicochemical Quality Determinations

Selective physicochemical parameters were determined according to Romanian standard [42] and Harmonized Methods of International Honey Commission [56]. All determinations were made in triplicate, and the results are presented as the average ± standard deviation. Water content was determined refractometrically using an Abbe digital refractometer (WYA-S Selecta Spain) and was expressed as mg/100 g. Electrical conductivity was measured at 20 °C in a 20% (*w*/*v*) honey solution in water with a KIT Consort conductometer (CONSORT nv, Belgium) and expressed as mS/cm, hydroxymethylfurfural (HMF) was determined spectrophotometrically according to White method (expressed as mg/kg) on a Varian Cary UV50 Multicell apparatus [56]. Sugar profile was determined by HPLC–IR [57], on a Shimadzu system equipped with an LC-10AD pump, DGU-14A degasser, SIL-10AV VP auto sampler and RID-10A refractive index detector. The separation column (Altima Amino 100 Å 5 µm, 250 mm × 4.6 mm), thermostated at 30 °C with a temperature controller, uses a mixture of acetonitrile/water as mobile phase with 1.3 mL/min flow rate in isocratic mode. Injection volume was 10 µL. Briefly, 5 grams of honey was weighted (0.001 g precision) and dissolved in 20 mL ultrapure water. The solution is transferred in a 100 mL volumetric flask containing 25 mL methanol. The final volume in brought to 100 mL with water. The sample is filtered through nylon syringe Millipore filter 0.45 µM and injected in HPLC.

To quantify the main sugars in honey, a calibration curve was built for each standard (fructose, glucose, sucrose) by injecting 7 mixtures of standards, where the concentration of fructose was 20–50 g/100 g, glucose was 10–40 g/100 g and sucrose was 0.3–15 g/100 g, with a regression coefficient of r^2^ = 0.9982. Results were expressed in g/100 g honey.

### 3.4. Pollen Analysis

Honey samples were subjected to pollen analysis according to Louvreaux et al. (1978) [58], Romanian honey quality standard requires 30, 25 and 20% *Robinia* pollen grains for superior, quality I and quality II honey and 40 and 35% *Helianthus* pollen for quality I and quality II honey, respectively [42]. The confirmation of botanical origin in the samples subjected to analysis was made according to these specifications. 

### 3.5. Total Polyphenol and Flavonoid Content

Total polyphenolic content was determined spectrophotometrically, following the Folin–Ciocâlteu method, which was modified for the identification of phenolic entities from honey solution [59]. Several determinations were made for identifying the optimal solvent for dissolving honey: distilled water, distilled water of pH = 2 (using concentrated HCl for pH adjustment) methanol, mixture of methanol:distilled water 1:1 (*v*:*v*); mixture methanol:distilled water of pH = 2, 1:1 (*v*:*v*). The criterion for choosing the best solvent was to obtain the highest absorbance, following the analysis of polyphenols by using the Folin–Ciocâlteu method. Solutions of honey (10 %, *w*/*v*)(0.5 mL) were mixed with 2.5 mL of Folin–Ciocâlteu reagent (diluted to 0.2 N with ultrapure water), and after 5 min, 2 mL of Na_2_CO_3_ solution (75 g/L in water) was added. The mixtures were placed in the dark for 2 hours and read at 760 nm towards a blank (same mixture without sample). The highest absorbance (corresponding to the most suitable solvent for the class of analyzed compounds) was obtained by diluting honey with methanol and mixture of methanol and distilled water of pH = 2. The calibration curve was made by using gallic acid (0–100 μg/mL), and the obtained r^2^ was 0.9980; the results were expressed as mg of gallic acid equivalents per 100 g of honey. 

Total flavonoid content was determined using two different methods, used in literature, adapted for honey analysis. First method was modified by Arvouet et al. (1994) [60] and adapted by Meda et al. (2005) [61] for honey analysis, methods that use as reagent a solution of AlCl_3_ in methanol. Five mL of honey solution (10%, *w*/*v*) was mixed with the same amount of aluminum chloride (2%, *w*/*v*) solution, and after 10 min, the absorbance was read at 415 nm by using a UV–VIS spectrophotometer, towards a blank containing 5 mL of honey solution and 5 mL of methanol. For the calibration curve, different dilutions of a 1 mg/mL quercetin in methanol (2.5–125 μg/mL) (*w*/*v*) were used to construct the calibration curve. The average of three independent readings represents the final value for every honey solution, and the results were expressed as mg equivalents of quercetin per 100 g honey. The second method for total flavonoids content was the Kim et al. (2003) method [7], adapted by Blasa et al. (2005) [62] for honey analysis method, using NaNO_2_, AlCl_3_ and NaOH as reagents. Briefly, 1 mL of honey solution (10%, *w*/*v* in appropriate solvent) was mixed with 0.3 mL of NaNO_2_ solution (5%, *w*/*v* in water), 0.3 mL AlCl_3_ (10%, *w*/*v* in methanol) and 2 mL NaOH (1 M). The absorbance was read at 510 nm towards a blank similarly prepared, replacing the sample volume with 1 mL methanol. Results were expressed as mg quercetin equivalents per 100 g honey.

### 3.6. Antioxidant Activity

Lately, different methods have been used to determine the antioxidant activity of biological samples [63,64,65]. Due to the fact that certain limitations are reported for every method, more than one single method is recommended to be made when performing a scientific study [66]. The simplest and the most used method for radical scavenging activity determination is the 1,1-diphenyl-2-picrylhydrazyl (DPPH) discoloration method. The dark blue color of DPPH* radical is decolored when it is reduced to hydrazine by reacting with different hydrogen donors, such as antioxidants. This ability is evaluated by using UV spectrophotometry, on the basis that the color intensity is indirect proportionally with the concentration of antioxidants and the reaction time, this change being followed at 517 nm. The method adapted for honey analysis [65] uses as positive control Trolox, an analogue of vitamin E. 

The free radical scavenging activity of the studied honey samples was determined by DPPH method. Antiradical activity may be expressed as follows:

% Inhibition = [(Absorbance_blank_ − Absorbance_sample_)/Absorbance_blank_] × 100 (1)

A calibration curve of different Trolox concentrations (0–0.025 mmols/L) was made and the percentage of inhibition for every concentration was determined. A calibration curve of % inhibition/Trolox concentration was constructed (r^2^ = 0.9990) in order to express the amount of antioxidants from every sample as milliequivalents Trolox/100 g honey. One milliequivalent (meq) is defined as its ability to reduce one milliequivalent of pro-oxidant. Briefly, 0.4 mL sample (0.1 mg/mL *w*/*v*) was mixed with 2.8 mL DPPH solution (0.03 mg/mL in methanol), and incubated in the dark for 30 min. The absorbance of DPPH solution left after the reaction with honey antioxidants was determined at 517 nm towards a blank. This procedure was repeated (same ratio of reagents) for different Trolox concentrations. 

Additionally, for all honey samples, the IC_50_ value (the concentration of honey that inhibits 50% of the DPPH^*^ radical) was determined and calculated. 

Different serial concentrations of honey were made, % inhibitions were determined and calibration curves of % inhibition/honey concentration were performed in order to calculate the IC_50_ value of every honey.

In the end, scavenging radical activity for different honey samples was expressed as % inhibition, milliequivalents Trolox/100 g honey and IC_50_.

Scientific studies have showed that the negative effect of environmental stress is mediated by generation of reactive oxygen species (ROS). The protection against ROS is given by endogenous antioxidants [67] or by the antioxidants from our diet [68]. Ferric Reducing Antioxidant Power (FRAP) method estimated directly the antioxidants from a sample, and is based on the ability of the analyte to reduce Fe^3+^ from the complex ferric tripiridil-tiazine (Fe^III^-TPTZ) to its ferrous form (Fe^2+^), with an intense blue color, which can be monitored by absorbance measurement at 593 nm. The method of measuring the reduction/antioxidant power (FRAP) was modified by Aljadi and Kamaruddin (2002) [69] and it was used in this study. The absorbance at 593 nm was read at 0 and 4 min of reaction, following incubation at 37 °C, on a calibration curve of different concentrations (0.1–1.0 mM) of FeSO_4_. The calibration curve absorbance/FeSO_4_ concentration was transformed into FRAP values (mM FRAP) by dividing the ΔA_593_ of the samples to ΔA_593_ of the standard multiplied by FRAP value of the 1 mM FeSO_4_ solution. The analysis was made on a BioTek Synergy HT spectrophotometer. Briefly, 10 μL of sample (0.1 mg/mL *w*/*v*) and 300 μL freshly prepared FRAP reagent (20 mL acetate buffer 300 mM, pH = 3.6; 2 mL TPTZ solution 10 mM; 2 mL solution FeCl_3_ 20 mM) were placed in microwell plates, including a control (10 μL ultrapure water and 300 μL FRAP) and standards for the calibration curve (different concentrations of FeSO_4_). The absorbance at 0 time (Ao) was registered at 593 nm. After incubation at 37 °C for 4 min, the absorbance was again read (A4), and a calibration curve of absorbance/FeSO_4_ concentration was made. Sample absorbencies were transformed into FRAP values (nM FRAP) by dividing the ΔA of the samples to ΔA of standard solution x FRAP value of 1 mM FeSO_4_ solution. The results were expressed as mM Fe^2+^/100 g honey.

### 3.7. Polyphenols (Flavonoids and Phenolic Acid) Isolation

A modified method of Ferreres et al. (1994) [70] was used for extraction of flavonoids and phenolic acid. Weighted honey sample (40 g) was dissolved with 5 parts of acidified water (adjusted to pH = 2 with HCl), on a magnetic stirrer, for about 30 min. The obtained solution was passed through a glass column containing Amberlite XAD-4 resin (Fluka Chemie, Germany). Phenolic substances from honey solution are bounded to Amberlite particles from the column, while sugars are washed with acidified distilled water (pH = 2) and in the end with neutral distilled water.

All phenolics (flavonoids and phenolic acids) were eluted from the column with ~400 mL methanol, collected in a round bottom flask and evaporated to dryness on rotary evaporator. The obtained residue was re-dissolved in 10 mL of distilled water and partitioned with ethyl acetate (4 × 20 mL) for the liquid extraction in a separation funnel. All resulting extracts were collected, evaporated to dryness, labeled and kept at −18 °C until analysis.

The partition with ethyl acetate was performed with a higher quantity of solvent than in the original method, for a complete extraction of phenolic compounds. Thus, the recovery percent of original standards of phenolic compounds, as well as the compounds from honey from the column, was situated between 83.09 and 98.08%. 

### 3.8. HPLC Analysis of Flavonoids and Phenolic Acids

For the flavonoid and phenolic acid determinations, a method described by Andjelkovic et al. (2008) [71], was used. The Agilent 1100 LC–MSD system consisting of vacuum degasser, quaternary pump, autosampler, DAD variable wavelength detector, 1100 6-port auto injector valve (20 µL loop) controlled by Agilent software v. A.09.03 (Agilent Technologies, Waldbronn, Germany), Phenomenex C18 (ODS, Octadecyl) security guard column, Phenomenex Luna C18 100 Å column (4.6 mm i.d. × 250 mm; particle size 10 µm), maintained at 35 °C. Flow rate of 1.0 mL/min was used for the mobile phase consisting in a mixture of 0.2% acetic acid in water (solvent A), methanol (solvent B) and acetonitrile (solvent C), in gradient mode (Supplementary Materials Table S1). Injection volume was 20 μL. Chromatograms were registered at 280 and 340 nm. The mass spectrophotometric measurements were made on an Agilent G1946D (SL) mass detector with an ion-trap mass spectrometer equipped with an electro spray ionization (ESI) system in negative ionization mode. Nitrogen at a pressure of 50 psi and a flow adjusted to 13 L/min was used as nebulizing gas. The full-scan mass spectra of the phenolic compounds were measured from *m*/*z* 100 up to *m*/*z* 1000. External standard method was used for the flavonoid and phenolic acid identification, where comparison of chromatographic data (retention times, UV spectra of compounds and MS fragmentation) with authentic standards is performed.

Calibration curves in the range 0.01–0.1 mg/mL of 25 standards of phenolic acids and flavonoids, with the regression coefficients between 0.9950 and 0.9999, were obtained. Results are expressed in mg phenolic compound/kg honey. 

Authentic markers’ retention times, maxima absorption, wavelength of the quantification and negative ESI–MS main fragments [M − H]¯ are presented in Supplementary Materials Table S2.

### 3.9. Statistical Analysis

Determinations of physicochemical analysis were made in series of three independent repetitions, and results were expressed as mean ± standard deviation (SD). Data were analyzed by one-way analysis of variance (ANOVA), followed by Tukey’s multiple-range test, using XLSTAT software (Addinsoft, New York, NY, USA). Furthermore, principal component analysis (PCA) and cluster analysis was performed by using the Paleontological Statistics (PAST) software [72]. Cluster analysis was performed on a Bray–Curtis similarity matrix, using complete linkage.

## 4. Conclusions

The amount and distribution of phenolic acids and flavonoids are affected by the floral origin in different honeys. All results were in accordance with Romanian and European regulations. The values obtained for physicochemical parameters are characteristic for these types of honey. The analysis of *Robinia* and *Helianthus* honeys originating from different locations in Romania showed that polyphenolic profile (phenolic acids and flavonoids) could be used as a complementary method for authenticity determination together with pollen analysis and other physicochemical analysis. High antioxidant activity was correlated with high phenolic content, as well as with specific identified phenols, confirming that phenolic acids in honey have a major contribution to its antioxidant and, thus, bioactive properties. One flavonoid compound (acacetin) from *Robinia* honey was proposed as a biochemical marker, based on different studies of monofloral honeys where this compound is absent. For *Helianthus* honey, it was confirmed that quercetin could be used as biochemical marker, together with a high content of luteolin. Abscisic acid was found in high amounts in samples where stress conditions (heat and humidity) are present. 

To determine more accurately the profile and concentration of the phenolic compounds from honey, one should use different extraction protocols, with a larger number of samples, in order to confirm the present results.

## Figures and Tables

**Table 1:** Main physicochemical characteristics for the black locust (*Robinia pseudoacacia*) and sunflower (*Helianthus annuus*) honey samples.

Sample	Moisture (%)	Electrical Conductivity (mS/cm)	HMF (mg/kg)	Glucose (%)	Fructose (%)	F/G Ratio	Sucrose (%)
RH-01	17.33 ± 0.12 ef	0.14 ± 0.01 ef	0.997 ± 0.03 g	29.86 ± 0.35 ef	42.29 ± 0.51 c	1.42 ± 0.02 c	0.70 ± 0.12 d
RH-02	17.83 ± 0.05 de	0.20 ± 0.00 d	0.18 ± 0.03 h	31.24 ± 0.56 ef	44.11 ± 0.22 a	1.41 ± 0.02 c	0.21 ± 0.10 fg
RH-03	17.37 ± 0.15 ef	0.15 ± 0.03 e	0.88 ± 0.06 g	33.0 ± 0.11 e	38.67 ± 0.50 e	1.70 ± 0.01 a	2.12 ± 0.23 a
RH-04	18.90 ± 0.10 a	0.10 ± 0.00 g	1.50 ± 0.04 f	31.93 ± 0.39 e	43.29 ± 0.38 b	1.35 ± 0.00 d	0.20 ± 0.02 g
RH-05	18.30 ± 0.10 c	0.18 ± 0.00 d	1.74 ± 0.04 de	29.66 ± 0.50 ef	42.53 ± 0.13 c	1.43 ± 0.02 c	0.35 ± 0.05 f
RH-06	17.43 ± 0.05 e	0.15 ± 0.00 ef	1.12 ± 0.10 g	31.16 ± 0.77 f	43.18 ± 0.25 b	1.38 ± 0.02 c	0.65 ± 0.03 de
RH-07	17.86 ± 0.11 de	0.14 ± 0.00 ef	1.38 ± 0.07 fg	31.98 ± 0.44 e	45.99 ± 1.54 a	1.43 ± 0.04 c	0.19 ± 0.06 g
RH-08	18.30 ± 0.10 c	0.19 ± 0.00 d	2.18 ± 0.32 cd	32.20 ± 0.24 e	40.06 ± 0.27 d	1.24 ± 0.01 e	0.35 ± 0.05 f
RH-09	18.63 ± 0.15 b	0.13 ± 0.00 f	2.04 ± 0.13 cd	28.86 ±0.31 g	42.96 ± 0.24 bc	1.48 ± 0.02 b	0.27 ± 0.04 f
RH-10	17.30 ± 0.10 f	0.14 ± 0.00 ef	1.85 ± 0.09 cd	30.19 ± 0.14 ef	39.51 ± 0.48 d	1.30 ± 0.01 e	1.63 ± 0.19 b
SFH-01	18.13 ± 0.20 de	0.56 ± 0.05 a	2.79 ± 0.59 c	44.08 ± 0.67 a	39.27 ± 0.23 d	0.89 ± 0.01 g	1.06 ± 0.15 c
SFH-02	18.36 ± 0.15 c	0.52 ± 0.02 a	4.29 ± 0.48 b	41.46 ± 0.52 c	39.26 ± 0.24 d	0.94 ± 0.00 g	0.03 ± 0.05 h
SFH-03	18.36 ± 0.41 c	0.28 ± 0.03 c	1.31 ± 0.33 fg	42.22 ± 0.35 c	38.88 ± 0.10 de	0.92 ± 0.00 g	0.06 ± 0.05 h
SFH-04	19.03 ± 0.40 a	0.26 ± 0.05 c	9.03 ± 0.51 a	44.96 ± 0.77 a	38.17 ± 0.30 e	0.85 ± 0.01 h	0.58 ± 0.03 e
SFH-05	17.83 ± 0.25 de	0.58 ± 0.02 a	5.15 ± 0.21 b	43.30 ± 0.61 b	38.31 ± 0.43 e	0.88 ± 0.00 g	0.77 ± 0.05 d
SFH-06	17.53 ± 0.41 e	0.46 ± 0.03 b	3.28 ± 0.19 c	41.87 ± 0.29 c	38.61 ± 0.27 e	0.92 ± 0.01 g	1.14 ± 0.17 c
SFH-07	18.06 ± 0.15 d	0.55 ± 0.02 a	1.53 ± 0.31 f	40.61 ± 0.37 d	39.49 ± 0.14 d	0.97 ± 0.00 f	1.41 ± 0.18 b
SFH-08	18.20 ± 0.30 cd	0.54 ± 0.04 a	1.82 ± 0.23 cde	40.11 ± 0.41 d	38.65 ± 0.39 de	0.96 ± 0.00 f	0.74 ± 0.21 d

RH-01–10—*Robinia pseudoacacia* honey; SFH-01–08—*Helianthus annuus* honey; HMF—hydroxymethylfurfural; F/G Ratio—fructose/glucose ratio. Values represent the average ± standard deviations of three independent determinations. Different letters within a column denote significant differences (*p* < 0.05).

**Table 2:** Total polyphenolic content, flavone/flavonols and total flavonoids in analyzed honey samples.

Sample Code	Total Polyphenols (mg GAE/100 g Honey)		Flavone/Flavonol Content (mg QE/100 g Honey)		Total Flavonoids Content (mg QE/100 g Honey)	
	MeOH	H_2_O:MeOH	MeOH	H_2_O:MeOH	MeOH	H_2_O:MeOH
RH-01	75.87 ± 0.50 k	60.19 ± 0.85 i	10.81 ± 0.53 fg	10.01 ± 0.10 ef	38.81 ± 0.30 e	42.49 ± 0.50 d
RH-02	82.35 ± 0.51 gh	75.18 ± 0.28 g	12.27 ±.26 f	10.51 ± 0.60 ef	36.14 ± 0.32 ef	41.88 ± 0.39 d
RH-03	76.44 ± 0.48 ij	72.32 ± 0.58 h	10.11 ± 0.27 g	10.12 ± 0.25 ef	33.48 ± 1.02 g	38.84 ± 0.50 e
RH-04	85.44 ± 0.76 g	83.87 ± 1.19 e	11.18 ± 0.25 fg	10.20 ± 0.29 ef	32.66 ± 0.47 g	36.85 ± 0.54 f
RH-05	83.69 ± 0.30 gh	79.77 ± 0.78 f	10.74 ± 0.50 fg	10.04 ± 0.11 ef	36.22 ± 0.39 ef	37.36 ± 0.44 ef
RH-06	76.65 ± 0.48 ij	72.32 ± 0.34 h	10.05 ± 0.18 g	9.37 ± 0.57 f	34.30 ± 0.89 fg	34.16 ± 0.42 gh
RH-07	82.30 ± 0.27 h	79.11 ± 0.55 f	11.27 ± 0.25 fg	9.85 ± 0.29 f	37.17 ± 0.28 e	35.53 ± 0.22 g
RH-08	77.91 ± 0.26 j	75.48 ± 0.51 g	9.81 ± 0.60 gh	8.94 ± 0.23 f	30.62 ± 0.54 h	32.71 ± 0.48 hi
RH-09	75.66 ± 0.45 k	71.33 ± 0.44 h	9.01 ± 0.50 h	7.81 ± 0.51 g	29.19 ± 0.27 h	31.31 ± 0.12 i
RH-10	80.52 ± 0.43 i	79.14 ± 0.35 f	10.16 ± 0.12 fg	10.15 ± 0.15 e	32.49 ± 0.47 g	33.82 ± 0.51 gh
SFH-01	134.21 ± 0.80 a	96.81 ± 0.44 c	34.13 ± 0.21 c	30.64 ± 0.63 c	46.55 ± 0.49 d	47.58 ± 0.54 c
SFH-02	98.75 ± 0.39 d	94.26 ± 0.97 c	31.06 ± 0.37 d	28.47 ± 0.19 d	48.33 ± 0.11 c	49.54 ± 0.03 b
SFH-03	95.79 ± 0.56 e	92.38 ± 1.59 cd	30.06 ± 0.18 e	28.10 ± 0.18 d	49.69 ± 0.49 b	50.55 ± 0.83 ab
SFH-04	99.32 ± 1.11 d	92.23 ± 1.87 cd	32.09 ± 0.13 d	30.02± ± 0.23 c	51.03 ± 0.39 ab	51.57 ± 0.60 a
SFH-05	115.78 ± 0.48 c	99.97 ± 1.61 b	36.13 ± 0.30 b	32.29 ± 0.36 b	52.33 ± 0.39 a	52.93 ± 0.68 a
SFH-06	92.40 ± 0.04 f	88.65 ± 0.53 e	30.20 ± 0.21 e	28.15 ± 0.13 d	48.96 ± 0.43 c	50.25 ± 0.40 b
SFH-07	98.79 ± 0.49 d	92.12 ± 0.34 d	34.13 ± 0.27 c	30.42 ± 0.21 c	50.15 ± 0.41 b	50.91 ± 0.43 ab
SFH-08	118.08 ± 0.55 b	107.55 ± 2.77 a	40.64 ± 0.40 a	35.97 ± 0.40 a	53.26 ± 0.42 a	53.56 ± 0.38 a

RH-01–10—*Robinia pseudoacacia* honey; SFH-01–08—*Helianthus annuus* honey; GAE—gallic acid equivalents; QE—quercetin equivalents; MeOH—Methanol; H_2_O:MeOH—water:methanol; values represent the average ± standard deviations of three independent determinations. Different letters within a column denote significant differences (*p* < 0.05).

**Table 3:** Radical scavenging activity (DPPH) and total antioxidant power (FRAP) for the analyzed honey samples.

Sample Code	Radical Scavenging Activity			Total Antioxidant Power
	% Inhibition	mmol Trolox/100 g Honey	IC_50_	FRAP Value (mM Fe^2+^)
RH-01	19.6 ± 0.70 ef	4.34 ± 0.15 ef	25.53 ± 0.93 ab	0.700 ± 0.02 ef
RH-02	20.7 ± 0.62 de	4.58 ± 0.14 de	24.17 ± 0.72 ab	0.730 ± 0.00 e
RH-03	19.9 ± 0.31 e	4.40 ± 0.07 de	25.17 ± 0.39 ab	0.709 ± 0.03 ef
RH-04	21.7 ± 0.70 d	4.80 ± 0.16 d	23.02 ± 0.75 b	0.766 ± 0.06 de
RH-05	17.9 ± 0.56 g	3.97 ± 0.12 g	27.95 ± 0.86 a	0.652 ± 0.00 h
RH-06	18.6 ± 0.21 fg	4.12 ± 0.05 fg	26.89 ± 0.32 a	0.670 ± 0.00 g h
RH-07	20.2 ± 0.25 de	4.46 ± 0.05 de	24.80 ± 0.31 ab	0.793 ± 0.04 d
RH-08	18.0 ± 0.35 fg	4.18 ± 0.08 fg	26.51 ± 0.49 ab	0.679 ± 0.02 gh
RH-09	19.2 ± 0.47 f	4.26 ± 0.10 ef	26.01 ± 0.65 ab	0.710 ± 0.01 ef
RH-10	19.9 ± 0.20 e	4.40 ± 0.04 de	25.13 ± 0.25 ab	0.770 ± 0.05 d
SFH-01	25.2 ± 0.55 ab	5.55 ± 0.12 bc	19.87 ± 0.44 cd	1.141 ± 0.17 bc
SFH-02	23.1 ± 0.75 c	5.11 ± 0.16 d	21.63 ± 0.70 c	0.991 ± 0.01 c
SFH-03	26.2 ± 0.25 ab	5.77 ± 0.05 b	19.11 ± 0.18 ef	1.249 ± 0.12 b
SFH-04	24.9 ± 0.45 b	5.50 ± 0.10 c	20.06 ± 0.36 cd	1.093 ± 0.06 c
SFH-05	27.1 ± 0.31 a	5.98 ± 0.07 a	18.43 ± 0.21 f	1.398 ± 0.04 a
SFH-06	25.9 ± 0.38 ab	5.70 ± 0.08 b	19.33 ± 0.28 de	1.173 ± 0.11 bc
SFH-07	24.6 ± 0.42 b	5.50 ± 0.09 c	20.06 ± 0.33 cd	0.972 ± 0.03 c
SFH-08	26.4 ± 0.42 ab	5.81 ± 0.09 ab	18.97 ± 0.30 ef	1.132 ± 0.11 bc

RH-01–10—*Robinia pseudoacacia* honey; SFH-01–08—*Helianthus annuus honey*; values represent the mean ± standard deviations of three independent determinations. Different letters within a column denote significant differences (*p* < 0.05).

**Table 4:** Identified phenolic acids and flavonoids in the analyzed black locust (*Robinia pseudoacacia*) honey samples.

Sample Code	Phenolic Acids (mg/kg Honey)					Abscisic Acid	Flavonoids (mg/kg)						Total
	*p*-hBA	VA	*p*-CouA	FerA	*t*-CinA		PinoB	Api	Kae	PinoC	Chr	Aca	
RH-01	0.38	nd	2.43	2.28	nd	2.95	2.19	1.42	0.59	0.38	0.95	1.15	9.46
RH-02	0.61	nd	nd	0.72	nd	15.91	2.28	nd	1.12	1.48	1.23	0.49	22.31
RH-03	nd	nd	nd	1.25	1.38	13.93	1.64	2.44	nd	0.56	0.69	1.14	21.03
RH-04	0.58	2.91	6.36	8.66	1.05	23.05	1.06	1.55	1.45	0.74	0.71	1.2	59.82
RH-05	0.75	nd	4.25	6.51	1.51	6.61	0.89	2.06	0.85	0.85	1.05	0.90	25.13
RH-06	0.25	nd	3.02	1.89	0.85	3.95	2.23	0.89	nd	1.20	1.24	1.75	12.13
RH-07	nd	nd	nd	4.25	1.40	15.45	1.90	nd	0.65	0.96	0.86	1.12	26.39
RH-08	nd	1.85	2.51	1.15	nd	7.45	2.12	nd	nd	1.26	0.98	0.95	17.77
RH-09	nd	nd	nd	6.02	nd	4.88	0.85	1.68	0.87	1.35	1.05	2.24	16.27
RH-10	nd	2.45	nd	2.45	nd	18.25	0.94	nd	nd	0.89	0.87	1.05	27.08
Average	0.26	0.72	1.86	3.52	0.62	11.24	1.61	1.00	0.55	0.97	0.96	1.20	24.55
SD	0.30	1.19	2.24	2.71	0.68	6.94	0.61	0.95	0.53	0.35	0.19	0.48	9.78

RH-01–10—*Robinia pseudoacacia* honey; nd—not detected; p-hBA—p-hydroxybenzoic acid; VA—vanillic acid; p-CouA—p-coumaric acid; FerA—ferulic aid; t-CinA—t-cinnamic acid; PinoB—pinobanksin; Api—apigenin; Kae—kaempferol; PinoC—pinocembrin; Chr—chrysin; Aca—acacetin.

**Table 5:** Identified phenolic acids and flavonoids in the analyzed sunflower (*Helianthus annuus*) honey sample.

Sample Code	Content of Phenolic Acids (mg/kg Honey)					Content of Flavonoids (mg/kg)								Total
	PrCatA	ChlA	*p*-hBA	CafA	*p*-CouA	Qe	Lut	PinoB	Api	Kae	PinoC	Chr	Gal	
SFH-01	3.00	1.63	2.07	2.08	3.09	1.21	27.09	2.13	1.97	0.54	1.44	0.69	1.53	48.47
SFH-02	3.74	2.37	nd	4.16	3.85	2.33	17.50	2.05	1.13	1.29	2.18	1.59	2.39	44.58
SFH-03	2.21	1.98	2.05	3.02	2.65	1.58	21.05	1.56	nd	0.83	1.05	0.56	1.52	40.06
SFH-04	1.98	2.15	1.79	2.56	2.96	1.35	18.75	1.68	1.24	1.14	1.67	0.95	1.96	40.18
SFH-05	2.5	1.86	1.96	3.05	1.98	1.98	22.78	2.10	2.02	1.04	2.01	1.28	1.49	46.05
SFH-06	2.95	2.21	nd	2.74	2.52	2.04	20.52	1.97	nd	0.75	1.89	0.84	2.07	40.50
SFH-07	2.64	1.58	nd	3.22	2.68	2.42	18.89	2.11	2.02	1.14	2.02	1.22	2.21	42.15
SFH-08	1.99	2.26	1.84	2.95	3.69	1.89	21.68	1.89	nd	0.98	2.01	1.49	1.98	44.65
Average	2.63	2.01	1.94	2.97	2.93	1.85	21.03	1.94	1.68	0.96	1.78	1.08	1.89	2.63
SD	0.60	0.29	0.12	0.60	0.62	0.44	2.99	0.21	0.45	0.24	0.38	0.38	0.34	0.60

SFH-01–08—*Helianthus annuus* honey; nd—not detected; PrCatA—protocatechuic acid; ChlA—chlorogenic acid; *p*-hBA—*p*-hydroxybenzoic acid; CafA—caffeic acid; *p*-CouA—*p*-coumaric acid; Qe—quercetin; Lut—luteolin; PinoB—pinobanksin; Api—apigenin; Kae—kaempferol; PinoC—pinocembrin; Chr—chrysin; Gal—galangin.

## Data Availability

Not available.

## Supplementary Materials

The following are available online. Table S1: HPLC–ESI–MS gradient used in polyphenolic determination. Table S2: Retention times and identification parameters for the standard compounds used in the study. Figure S1: Chromatogram of *Robinia pseudoacacia* honey. Figure S2: Chromatogram of *Helianthus annuus* honey. Figure S3: UV spectra of quercetin standard, identified compound and MS fragmentation ions.

## Sample Availability

Not available.
