# Supplementary material for: Bioactive Molecules for Discriminating Robinia and Helianthus Honey: High-Performance Liquid Chromatography–Electron Spray Ionization–Mass Spectrometry Polyphenolic Profile and Physicochemical Determinations
Source: Molecules. 2021 Jul 22;26(15):4433. doi: 10.3390/molecules26154433 (PMC8347174; doi:10.3390/molecules26154433)
Supplement: Supplementary file 1 [file molecules-26-04433-s001.zip › molecules-1311444-supplementary.pdf]

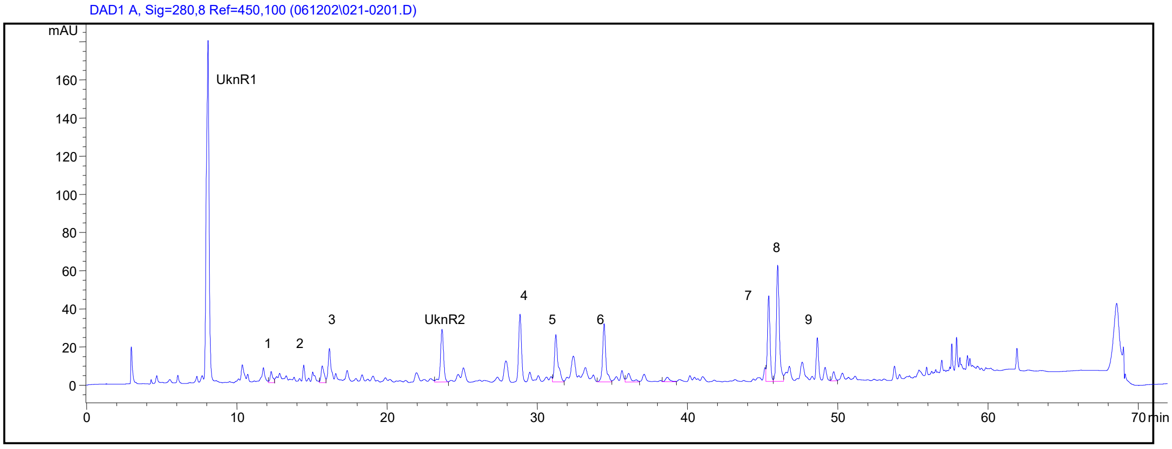

Figure S1. LC-MS chromatogram of phenolic acids and flavonoids in *Robinia pseudoacacia* (black locust) honey (sample RH-06-05). Separation conditions are described in the text. Peak identification: $UknR_1$ - Unknown *Robinia* 1; 1- p-hydroxybenzoic acid; 2 - Ferulic acid; 3 - t-cinnamic acid; 4 – Abscisic acid; $UknR_2$ – Unknown *Robinia* 2; 5 – Pinobanksin; 6 – Apigenin; 7 – Pinocembrin; 8 – Chrysin; 9 – Acacetin.

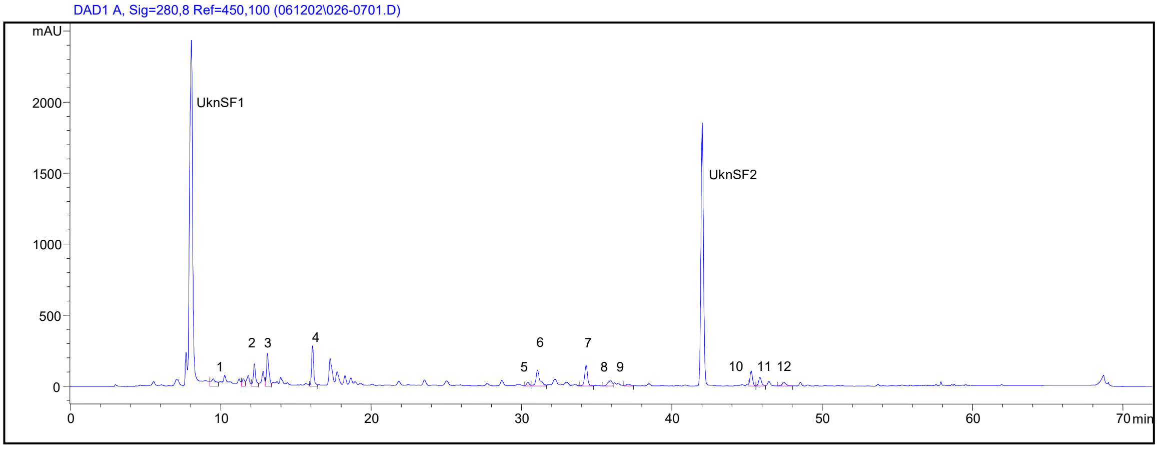

Figure S2. LC-MS chromatogram of phenolic acids and flavonoids in *Helianthus anuus* (sunflower) honey. Peak identification: $UknSF_1$ – Unknown Sunflower 1; 1 – Protocatecuic acid; 2 – Chlorogenic acid; 3 – Caffeic acid; 4 – p-coumaric acid; 5- Quercetin; 6 – Luteolin; 7 – Pinobanksin; 8 – Apigenin; 9 – Kaemferol; $UknSF_2$ – Unknown Sunflower 2; 10 – Pinocembrin; 11– Chrysin; 12 – Galangin.

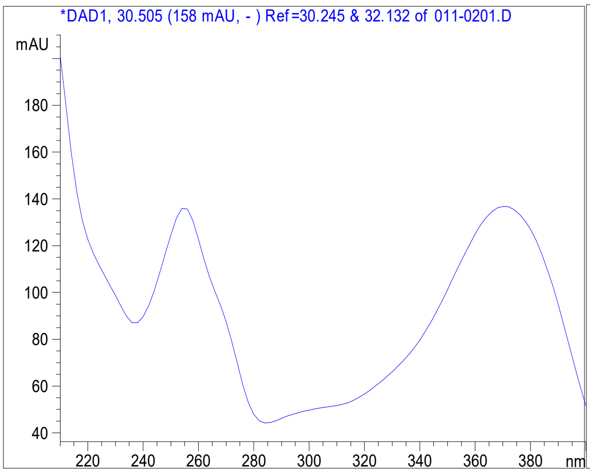

A

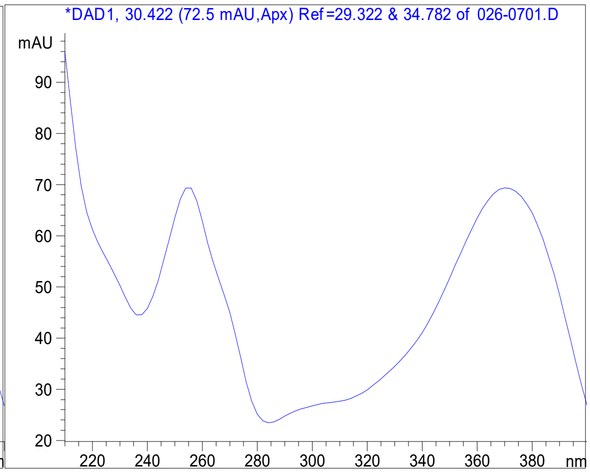

B

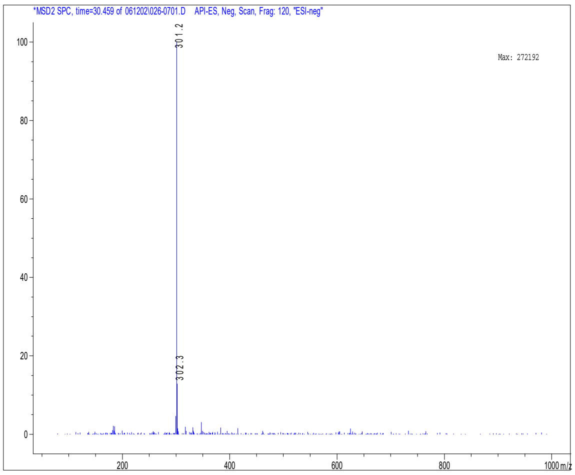

C

Figure S3. Quercetin identification : A – UV-VIS standard spectra ; B – UV-VIS compound spectra ; C – Electrospray ionisation mass spectra (negative ionization).

Table S1. Retention times and identification parameters for the standard compounds

Standard compound	R_T (min)	Quantification λ (nm)	ESI-MS* $[M-H]^-$	UV Bands (nm)
Protocatecuic acid	9.68	280	153	255, 289
Catechin	11.34	280	289	280
Chlorogenic acid	11.94	340	353	218, 240, 295^{sh}, 324
p-hydroxybenzoic acid	12.43	280	137	254, 273, 285^{sh}
Caffeic acid	13.30	340	179	218, 242, 298^{sh}, 326
Vanillic acid	13.33	280	167	218, 260, 290
Syringic acid	13.69	280	197	216, 275
Homovanillic acid	13.82	280	181	230, 280
Vanillin	15.67	280	151	279, 310
p-coumaric acid	16.37	280	163	212, 226, 300^{sh}, 310
Ferulic acid	17.56	280	193	206, 218, 234, 296^{sh}, 322
Naringin	20.14	340	580	284, 340^{sh}
Rosmarinic acid	23.15	340	359	220, 235^{sh}, 248^{sh}, 290, 330
Miricetin	23.30	340	317	255, $305^{sh,}$ 375
Abscisic acid	29.14	280	263	240^{sh}, 265
Quercetin	30.82	340	301	254, 270^{sh}, 305^{sh}, 367
Luteolin	31.07	340	285	255, 267^{sh}, 292^{sh}, 350
t-cinnamic acid	31.82	280	147	216, 272
Apigenin	36.28	340	269	268, 300^{sh}, 335
Kaemferol	36.37	340	285	267,294^{sh}, 325^{sh}, 365
Isorhamnetin	37.54	340	315	253, 372
o-coumaric acid	42.64	280	163	215, 226^{sh}, 280, 330
Pinocembrin	45.52	280	255	230^{sh}, 290, 335
Chrysin	45.89	280	253	245^{sh}, 270, 335
Acacetin	46.93	340	283	265, 335
Galangin	47.72	340	269	240^{sh}, 265,310, 365

sh - shoulder
